# Identifying Risk Factors for Autos and Trucks on Highway-Railroad Grade Crossings Based on Mixed Logit Model

**DOI:** 10.3390/ijerph192215075

**Published:** 2022-11-16

**Authors:** Lan Wu, Qi Shen, Gen Li

**Affiliations:** College of Automobile and Traffic Engineering, Nanjing Forestry University, Nanjing 210037, China

**Keywords:** injury severity, vehicle type, grade crossings, mixed logit model

## Abstract

This study aimed to determine different influencing factors associated with the injury outcomes of heavy vehicle and automobile drivers at highway–rail grade crossings (HRGCs). A mixed logit model was adopted using the Federal Railroad Administration (FRA) dataset (*n* = 194,385 for 2011–2020). The results show that drivers’ injury severities at HRGCs are enormously different between automobile and truck/truck–trailer drivers. It was found that vehicle speed and train speed significantly affect the injury severity in automobile and truck drivers. Driver characteristics such as gender and driver actions significantly impact the injury severity in automobile drivers, while HRGC attributes such as open space, rural areas, and type of warning device become significant factors in truck models. This study gives us a better understanding of the differences in the types of determinants between automobiles and trucks and their implications on differentiated policies for car and truck drivers.

## 1. Introduction

In the United States, traffic accidents are common. For example, there were six million accidents and 37,461 fatalities in 2016 [1]. HRGC accidents often result in incredibly serious losses when involved in traffic accidents because the weight of a train is usually 400 times that of an automotive vehicle [2].

**Figure 1:**
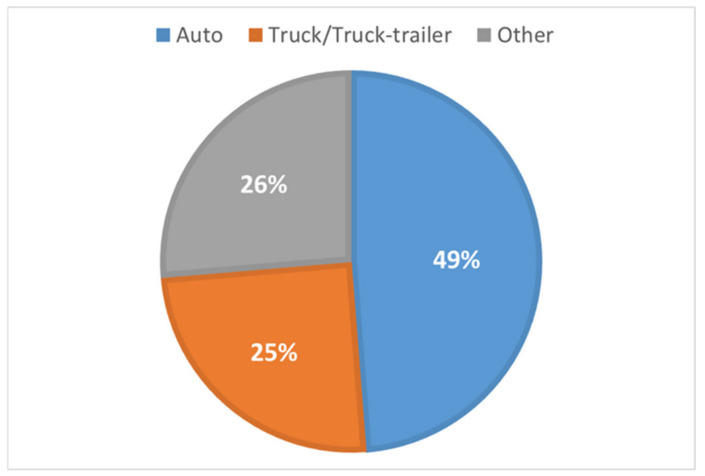
Accidents by vehicle type at HRGCs.

Table 1 presents the details of all of the vehicle types involved in crashes from 2011 to 2020. Although heavy vehicles only make up about 4% of all vehicles on the road [3], according to FRA crash data, heavy-vehicle (truck/truck–trailer) crashes at HRGCs accounted for 24.9% of all HRGC-related crashes reported from 2011 to 2020. As shown in Figure 1, automobile accidents account for the largest proportion, reaching 48.8%. It is important to investigate crashes involving trucks and automobiles at HRGCs because of the high proportion of accidents and the huge differences in vehicular characteristics such as length, weight, and stopping distance [4] as well as in the acceleration characteristics of these two types of vehicles.

Many researchers have only investigated truck crashes or trucks as one of the influencing factors related to injury severity. The current study fits in with studies using statistical modeling methods to analyze accidents involving trucks. Yu et al. [5] found that huge differences existed in the factors related to injury outcomes in accidents involving trucks in work zones on rural and urban highways. Zhu and Srinivasan [3] discussed potential factors that influence injury severity in accidents involving trucks. These factors included the time of the day, the day of the week, and driver behaviors such as drinking or getting lost. F. Chen and Chen [6] applied mixed logit models to analyze truck accidents on rural highways. Data were classified into two groups: single- and multiple-vehicle accidents. The results indicated that the models achieved higher accuracy when snowy weather and light traffic conditions were considered random parameters in both single-vehicle and multiple-vehicle models. Khorashadi et al. [7] studied injury severity in large truck accidents in rural and urban areas. Rural and urban areas both use multinomial logit models. Both driver and vehicle characteristics were found to play important roles in accidents. However, not all variables were significant in these two models. The authors attributed this to the differences in the demands placed on driver characteristics. Hao and Daniel [8] explored influencing factors related to injury severity at HRGCs. An OP model was used in the study. The research selected data from 2002 to 2011. Siti et al. [9] evaluated the risk of heavy vehicles passing through intersecting areas using Petri nets. The results showed that factors such as traffic level of service (LOS), crossing distance, and the percentage of heavy vehicle were significant. Khattak and Gao [10] found that violations increased with more truck traffic and longer time between train arrivals based on the frequency and type of crossing gate, and this was associated with the violations of truck drivers. Hao et al. [11] investigated factors related to the severity of the injuries incurred to truck driver at HRGCs utilizing data from the FRA database from 2002 to 2011 using an ordered probit model. According to data analysis results, factors such as high speed, bad weather, and visibility were positively correlated with the injury severity of truck drivers. Truck driver behavior and environmental factors were also significant. Fan et al. [12] developed an ordered-response logit model. The results showed that driving a truck–trailer decreased the likelihood of injury and fatal crashes. Companies assigning excessive work to truck drivers is a major cause of accidents [13]. There is little research on automotive vehicles, and most studies select automotive vehicles as a reference or base group due to them being present in large amounts. Hao et al. [14] selected automotive vehicles as a base group to investigate the effects of age and gender on the injury severity resulting from motor vehicle accidents at HRGCs. The results of the study indicated that young drivers lack experience, while older drivers suffer from long reaction times.

Besides the focus on contributing factors related to injury severity at HRGCs, selecting suitable statistical models to determine these factors is also important. Ordered probit [15,16], ordered logit [17,18], multinomial logit [19,20], and mixed logit [21,22] models have been widely adopted to determine injury severity in the past decade. Haleem [22] compared a mixed logit model with the binary logit model to investigate the determinant factors that result in accidents at private HRGCs. The result showed that the mixed logit model obtained a more realistic result. Ye and Lord [23] researched how to quantify the sample-size requirements for crash severity modeling using three methods: an ordered probit model, a multinomial logit model, and a mixed logit model. The mixed logit model was found to outperform the multinomial logit model, but it required a larger sample size than the other two models. Zhao and Khattak [24] compared an ordered probit model, multinomial logit model, and mixed logit models to conduct an investigation on the factors associated with injury severity levels in motor vehicle drivers involved in accidents at HRGCs. It turned out that the mixed logit model and multinomial logit model were more suitable for injury severity analysis. The reason why mixed logit models are better than multinomial models is because multinomial models require the irrelevant alternative (IIA) property to be independent. However, no studies have indicated whether or not the IIA property was met. Liu and Khattak [25] found that gate-violation behaviors have a spatial correlation with injury severity. This finding suggests that a mixed effect model should be applied to HRGCs crashes [26,27]. At the same time, machine learning methods [28,29] have started to be used in injury severity research.

Previous studies have focused on interpreting the crashes involving special vehicle types such as trucks, motorcycles, bikes, or pedestrians, by taking automotive vehicles, the largest proportion of vehicles, or all the other vehicle types as a reference group. As a result, relatively few studies have conducted comprehensive analyses on automotive vehicle crashes at HRGCs. Automobiles and trucks are both important in this study. Moreover, the differences in the size, speed, and quality of truck and automobiles are obvious, but the differences between truck and automobile drivers are often overlooked. Truck drivers, as a profession, usually receive formal training, such as accident response training, mandatory entry-level training, and maintenance training. Truck drivers who have received formal training are significantly less likely to crash [30]. Apparently, it is unreasonable to apply the same policy to automobile and truck/truck–trailer drivers due the differences in vehicle performance but also in the drivers themselves. Variables such as the position of highway users and the actions of highway users were taken into consideration to reflect this heterogeneity. This study aims to estimate the effect of various factors on the level of injury severity classified according to two vehicle types at HRCGs in the United States using data from the FRA database from 2011 to 2020. In light of previous research, a mixed logit method is adopted in this study.

## 2. Materials and Methods

### 2.1. Data

#### 2.1.1. Data Source

The dataset came from the FRA HRGC inventory and crash database. This section describes the details of the dataset and the data-reduction methods.

The original dataset covers the period from 2011 to 2020 and consists of 194385 HRGC crashes. The dataset is made up of two major components: HRGC history files and HRGC inventory files. The unique ID representing each crossing among the two files connects them. HRGC files possess information such as accident time, vehicle and train type, the behavior of highway users, weather on the day of the accident, and drivers’ genders, etc. HRGC inventory files contain information such as crossing attributes, AADT, type of control device, geometric characteristics, etc. These two files complement each other and form the completed database.

#### 2.1.2. Data Reduction

Automotive vehicles make up about 48.8% of the total number HRGC crashes. Table 1 shows the exact number of reported crashes and the injury severity levels of each accident. Missing values for variables and some mismatched crossing IDs were excluded from the dataset. The final dataset with complete information for model estimation contains data for 3262 heavy-vehicle and 7427 auto-vehicle accidents.

#### 2.1.3. Data Description

Injury severity considering PDO, injury, and fatal crashes is the dependent variable in this study. The automobile dataset consists of 589 (7.9%) fatal crashes, 2277 (30.7%) injury crashes, and 4561 PDO (61.4%) crashes. The application of the mixed logit model requires the selection of a base category. The category ‘PDO’ was selected as the base category because it has the highest proportion of crashes. The heavy vehicle dataset consists of 160 (4.9%) fatal crashes, 696 (21.3%) injury crashes, and 2406 (73.8%) PDO crashes. The independent variables in this study include driver attributes, geometric HRGC characteristics, types of controls (active and passive), train speed, etc. Table 2 presents the details of some of the variables used for model estimation. Variables with strong correlations were filtered out in this study. A total of 25 variables were taken into consideration. However, not all variables were significant for automobile and truck/truck–trailer model estimation owing to the different attributes of these two types of vehicles.

Some variables might lead to unclear understanding. These variables are described in detail below:

(1) Truck indicator in crash: Trucks as a percentage of heavy vehicles.
(2) Position of highway users: drivers who are stalled or stuck in the crossing; the position where drivers would like to go is blocked or the driver is blocked by external factors.
(3) Position of highway users: drivers who are stopped in the crossing area due to their own free will.

### 2.2. Method

The mixed logit model is not limited to the IIA property, which differs from the multinomial logit model [31]. IIA property states that all pairs of alternatives are equally not correlated, which obviously contradicts the real situation. The mixed model is developed from the multinomial logit model, which addresses a weakness of multinomial logit limited to IIA property by allowing parameter values to vary across observations [32]. In other words, the multinomial logit is not able to account for the heterogeneity due to correlation between variables. A model that fails to explain unobserved heterogeneity can lead to unrealistic conclusion [33,34]. The study aims to predict the probability of the level of injury in highway-rail grade crossing accidents based on data with discrete outcomes of driver injury severity of PDO, injury, and fatality. As a result, the mixed model was selected for this study.

The utility function that determines the driver injury severity is as follows [35,36]

(1) $$T_{in}=\beta_{i}X_{in}+\varepsilon_{in},$$

where $T_{in}$ is a linear function which represents the injury severity level i for HRGCs crashes n. $X_{in}$ is a vector of independent variables for severity outcome of level i for *n*th highway-rail grade crossing accident. $\beta_{i}$ is a vector of uncertain coefficients to be determined and $\varepsilon_{in}$ is an error term for random noise.

The basic form of the multinomial logit model, with (optional) alternative specific constants $\partial_{j}$ and attributes $X_{ij}$,

(2) $$P_{i}(j)=\frac{\exp(\partial_{j}+\beta_{l}X_{ij})}{\sum_{j=1}^{J}\exp(\partial_{j}+\beta_{l}X_{ij})},$$

where $P_{i}(j)$ = the probability of *i*th accidents turning into discrete injury severity level j. To address the problem of unobserved heterogeneity, the mixed logit model allows $\beta_{i}$ for varying across all the observations. So developed $P_{i}(j)$ is:

(3) $$P_{i}(j)=\int P_{i}(j)f(\beta_{i}\left|\varphi )\right.d\beta_{i},$$

Random parameters are introduced with $f(\beta_{i}\left|\varphi \right.)$, where φ is a vector of variables of the chosen density function (mean and variance). By putting the values of Equation (2) in Equation (3), we get [35]

(4) $$P_{i}(j)=\int \frac{\exp(\partial_{j}+\beta_{l}X_{ij})}{\sum_{j=1}^{J}\exp(\partial_{j}+\beta_{l}X_{ij})}f(\beta_{i}\left|\varphi )\right.d\beta_{i},$$

where $P_{i}(j)$ is the probability of injury severity i conditional on $f(\beta_{i}\left|\varphi )\right.$. Under the circumstance of $f(\beta_{i}\left|\varphi )\right.=1$ the model reduces to simple MNL. If the variance in *φ* is determined to be significantly different from zero, there will be accident-specific variations of the effect of X on injury severity across each crash observation n, with the density function $f(\beta_{i}\left|\varphi )\right.$ used to determine the values of $\beta_{i}$ across crashes [37].

In order to find the optimal fitting performance of the three models, we introduce the Akaike Information Criterion (*AIC*), Bayesian Information Criterion (*BIC*), and the value of the likelihood function (*LL*) as evaluation indicators. Lower *AIC*, *BIC* value, and a higher *LL* value indicate a better model.

(5) AIC=2N−2IN(L),

(6) $$BIC=IN(N_{1})\cdot N-2IN(L),$$

(7) $$LL=\sum_{i=1}^{I}\sum_{j=1}^{J}\delta_{ij}\cdot IN(P_{ij}),$$

where N is the number of parameters; L is the value of the likelihood function; $N_{1}$ is the number of observations; $\delta_{ij}$ is the indicator variable, when the driver i is at the injury severity level of j, the variable $\delta_{ij}$ equals 1, otherwise, equals 0.

## 3. Results

All independent variables obeyed normal distribution by default and were tested as random variables one by one. The parameters remained random during model estimation if they were statistically significant at a 90% significance level at least. The estimation selected Standard Halton Sequence (SHS) intelligent draws. The study set 300 as the number of draws (SHS) and as the maximum number of iterations because model accuracy no longer improves after 300 runs.

Table 3, Table 4, Table 5 and Table 6 show the results of the mixed logit estimation based on two types of vehicles. In the final estimations for the truck model, the indicator for intersecting roadways within 500 feet at an HRGC was found to be a random parameter (denoted with random), which means that the variable may vary across accidents. The rest are all fixed parameters. In the final estimation of the automobile model, the indicator for the general profiles of the drivers followed a normal random distribution and proved to have the best-fitting distribution among the other indicators in the injury severity models [38,39].

### 3.1. Truck/Truck-Trailer Model

The results revealed that 12 parameters were statistically significant in highway–rail grade crossing accidents involving trucks. Vehicle speed and train speed were statistically significant at the 90% and 99% significance levels, maintaining a positive correlation. These findings conform to former studies indicating that higher speed is one of the most important factors contributing to more severe injuries [40]. Crashes involving trucks at highway–rail grade crossings obviously have a higher chance of a more severe crash. Crashes involving trucks accounted for 8.4% of fatality and 32.4% of injury accidents; however, while there was a higher number of truck–trailer accidents (72%), there were had lower percentages of fatality (3.5%) and injury (17%) accidents. Fewer truck accidents but a higher rate of fatalities explains the positive significance associated with the truck indicator for the fatal accident category in the severity levels. One potential reason may be that trailers can be removed from the truck trailer. Thus, it is easier for the driver of a truck–trailer to separate from the cargo, while the truck’s integrity might put the driver in danger when an accident happens.

Turning to HRGCs attributes, variables such as crossings in rural areas, open space, intersecting roadways within 500 ft of a crossing, primary obstruction of track view, and an audible warning device were statistically significant. Crashes in rural areas tended to be more severe. There were 2114 accidents reported in rural areas, accounting for 64.8%. Of these crashes, fatal crashes comprised 23.1%, and injury crashes comprised 5.6%. At the same time, driving in an open space increased the probability of injury accidents by 10.2%. These two facts can be explained by drivers potentially driving more casually in open space/rural areas because of the low-density population in rural areas, which leads to more open space and a lack of advanced control devices. Primary obstruction of track view was negatively associated with the severity level of injuries. A blocked track view reduces the possibility of injury by 2.8%. Conditions that obstruct track view include passing trains, standing rail road equipment, permanent structures, and highway vehicles. In other words, it is easier for the drivers to perceive that there is an HRGC when the track view is blocked. Consequently, the driver will slow down or stop immediately because of the obstruction, even if the driver does not see tracks. Intersecting roadways within 500ft of crossings increases the possibility of injury by 9.8%. The reason for this may be that more intersecting roadways increase traffic volume and limit the sight distance. Both complicate the situation at HRGCs. Audible warning devices for active control were examined during model estimation. In highway–rail grade crossing accidents, the probability of injury decreased by 11% for crossings with an audible warning device. This finding is consistent with the findings of a previous study [41].

It is shown in Table 3 that driver attributes that have a tendency to increase the possibility of fatality include driver age above 55 and going around gates. As a result of neglecting active control, drivers are more prone to fatal accidents. Crash-specific characteristics correlating positively with injury severity level were when trains struck road users and vehicles moving over the crossing. These two characteristics appear reasonable and consistent with each other. There is no doubt that a vehicle is fragile when struck by a train due to the weight and size of the train. About 64% of total crashes (2011–2020) were the result of a road user moving over the crossing, of which 27.1% were injury crashes, and 6.7% were fatal crashes.

### 3.2. Auto Model

HRGC attributes include rural areas and warning devices: stop signs were found to be statistically significant at the 99% significant level, and both rural areas and warning devices were positively associated with injury severity. Consistent with the truck model, rural areas were found to increase the probability of fatal accidents by 27.8% and the probability of injury accidents by 8.4%. The reason was the same: drivers may drive more aggressively, an example of such behavior being driving at high speeds in an open area. The probability of fatality increases by 5.6% when crossings are equipped with a stop sign. In line with previous studies, crossings with only passive control measures such as stop signs were positively correlated with high-level injury severity, something proven in this research.

As we can clearly tell from the results in Table 5 and Table 6, the automobile model has more driver characteristics than the truck model. Table 5 indicates that drivers older than 55 years old are more likely to experience a serious accident, with an 18.5% increase in the probability of a fatality. One reason may be that older drivers (above 55 years of age) have decreased visibility and reaction time. Turning to the effect of driver gender, the total number of crashes involving female drivers comprised 8.2% fatal and 33.7% injury crashes, whereas the total number of accidents involving male drivers was higher (57.2%), but there were lower percentages of fatal and injury crashes: 7.7% and 28.4%, respectively. In terms of driving behavior, behaviors such as stopping and then proceeding and stopping at a crossing have a negative correlation with injury severity level, decreasing the probability of a fatal accident by 5.7% and 1.1%, respectively. Consistent with previous research, appropriate driving behavior reduces the risk of accidents [27]. Both of the behaviors mentioned above give drivers more time to brake and react to the oncoming train. However, aggressive driving behaviors such as going around crossing gates and not stopping increase the probability of a fatality by 9.6% and of an injury by 4.4%. Crash-specific characteristics such as vehicles going over the crossing increase the likelihood of drivers experiencing a higher level of injury severity. Meanwhile, being stalled or stuck on a crossing was negatively associated with injury severity, decreasing the probability of a fatality by 25%. Both findings appear reasonable and consistent with findings related to driver behavior. About 62.1% of the total crashes were reported to have occurred when a road user was moving over the crossing accompanied by the behavior of going around/through the crossing gates. Additionally, being stalled or stuck on a crossing has an inevitable connection with behaviors such as being stopped on a crossing.

A vehicle driving speed over 50 mph increases the risk of fatality by 1.6%. Consistent with the truck model and previous studies, high speeds always lead to an increase in the injury severity level. More severe accidents may occur when drivers do not give themselves enough time to react and brake when a train is coming. Meanwhile, it is shown in Table 5 that a train speed of more than 50 mph increases the likelihood of a fatality by 16.8% at HRGCs. As a result, a policy to limit train speed is of great importance for safety, as reducing train speed gives more time for break action and reduces the impact from trains hitting vehicles.

## 4. Discussion

According to the results of two mixed logit models, many factors effect injury severity. These variables can be divided into two groups: subjective factors (driver) and objective factors (road and environment). Among the subjective factors (with the exception of speed), the auto model had nine significant factors, while the truck model only had three significant factors. Meanwhile, the truck model had two more objective factors than the auto model.

Hence, comprehensive and targeted countermeasures should be applied to decrease the injury severity level. First of all, speed limit measures both for vehicles and trains driving at speeds higher than 50 mph, such as fines for speeding, speed monitoring, and warning signs for the speed limit, are recommended. Priority should be placed on controlling train speed due to it having a greater impact on injury severity than vehicle speed in both the automobile and truck models. Secondly, policy recommendations accounting for the differences in trucks and autos are as follows: Regarding driver characteristics, the automobile model had nine variables, more than the three variables in the truck/truck–trailer model. Compared to automobile drivers, most truck drivers are professional drivers, which means that they receive more professional training. On the one hand, automobile drivers should receive more safety education when engaging in dangerous driving behaviors such as going around crossing gates. On the other hand, drivers older than 55 years old should take occasional breaks, especially during long drives, to avoid suffering from slow reaction times and fatigue while driving. With regard to trucks, the administration should pay more attention to completing warning devices because there were significantly more highway–rail grade crossing attributes that affected the truck model than the automobile model. The addition of active control measures such as gates, audible signals, and flashing lights at HRGCs only equipped with passive control measures such as stop signs should be prioritized, especially in rural areas. Finally, HRGCs with a sufficient amount of traffic signals and proper visibility are obviously more secure, demanding thoughtful crossing design before construction.

## 5. Conclusions and Future Works

This paper explores the potential factors that influence injury severity levels in HRGC accidents involving two vehicle types: trucks/truck–trailers and automobiles, and compares those factors.

Separate models for accidents involving trucks and automotive vehicles were developed, and the model results were compared to identify the differences between these two types of crashes. Model estimation indicates that vehicle and train speed have a strong effect on the injury severity level in HRGC accidents involving trucks and automobiles. Hence, the implementation of speed limits could significantly improve safety. In terms of truck models, driving in open spaces and rural areas result in a higher likelihood of severe injury, while audible warning devices for active control decrease the probability of injury accidents. As a result, enforcement efforts aimed at equipping highway–rail grade crossings with active measures should be taken. Similar to the automobile model, drivers of trucks/truck–trailers going around crossing gates have an increased likelihood of experiencing a severe injury in an accident. However, appropriate behaviors such as stopping and then proceeding, or stopping at the crossing could decrease the likelihood of injury in automobile drivers. Consequently, administrators should pay more attention to the education and management of automobile drivers. Last but not least, both truck and automobile drivers who are older than 55 years of age should consider taking public transportation due to their higher risk of being involved in crashes.

This study has some limitations. First, considering the temporal effects of data and selecting data that are more valuable to the present would make the study more convincing. In addition, models that can capture the correlation between variables should be applied in future studies. Last but not least, geometric properties not included in FRA database such as sight distance and road width could be collected by Geographic Information Science (GIS) to provide more useful information.

## Figures and Tables

**Table 1:** Injury Severity of Drivers in Crashes at HRGCs (2011–2020).

Type of Vehicle	Total Reported Crashes	Fatal	Injury	Property Damage Only (PDO)
Auto	9477	6331	2524	622
Truck	1392	916	382	94
Truck-trailer	3450	2831	516	103
Pick-up truck	2631	1643	727	261
Van	562	344	164	54
Bus	34	33	1	0
School bus	8	6	1	1
Motor cycle	67	23	26	18
Other veh	1210	729	360	121
Pedestrian	30	2	7	21
Other	577	261	161	155
Total	19,438	13,119	4869	1450

**Table 2:** HRGCs accident characteristics.

		Auto		Truck/Truck-Trailer	
Description		Frequency	Percentage	Frequency	Percentage
Dependent Variable					
Driver	PDO	4667	62.8%	2406	73.8%
	injured	2177	29.3%	696	21.3%
	fatal	583	7.8%	160	4.9%
Independent Variables					
Vehicle speed	1 (more than 50 mph)	119	1.6%	43	1.3%
	0 (less than 50 mph)	7308	95.2%	3219	98.7%
Train speed	1 (more than 50 mph)	894	12%	464	14.2%
	0 (less than 50 mph)	6533	88%	2798	85.8%
Truck indicator in crash	1 (truck)	/	/	913	28%
	0 (truck-trailer)	/	/	6514	72%
Position of highway user: stalled or stuck on crossing	1 (yes)	1098	14.8%	475	14.6%
	0 (no)	6329	85.2%	2787	85.4%
Position of highway user: stopped on crossing	1 (yes)	1701	22.9%	699	21.4%
	0 (no)	5726	79.1%	2563	78.6%
Position of highway user: moving over crossing	1 (yes)	4619	62.2%	2088	64%
	0 (no)	2808	27.8%	1174	36%
Position of highway user: trapped on crossing by traffic	1 (yes)	3	0%	/	/
	0 (no)	7424	100%	/	/
Position of highway user: blocked on crossing by gates	1 (yes)	6	0%	/	/
	0 (no)	7418	100%	/	/
Circumstance of accident	1 (rail equipment struck highway user)	5755	77.4%	2935	90%
	0 (rail equipment struck by highway user)	1672	22.6%	327	10%
Action of highway user: went around the gates	1 (yes)	1050	14.1%	183	5.6%
	0 (no)	6377	85.9%	3079	94.4%
Action of highway user: stopped and then proceeded	1 (yes)	418	5.6%	274	8.4%
	0 (no)	7009	94.4%	2988	81.6%
Action of highway user: did not stop	1 (yes)	2599	35%	1514	46.4%
	0 (no)	4828	65%	1748	53.6%
Action of highway user: stopped on crossing	1 (yes)	2027	27.3%	845	25.9%
	0 (no)	5400	82.7%	2417	74.1%
Action of highway user: other	1 (yes)	889	12%	394	12.1%
	0 (no)	6538	88%	2868	87.9%
Action of highway user: went around/thru temporary barricade	1 (yes)	30	0.1%	2	0.1%
	0 (no)	7297	99.9%	3260	99.9%
Action of highway user: went thru the gate	1 (yes)	352	4.7%	50	1.5%
	0 (no)	7075	95.3%	3212	98.5%
Action of highway user: suicide/attempted suicide	1 (yes)	82	1.1%	/	/
	0 (no)	7345	98.9%	/	/
Primary obstruction of track view	1 (obstructed)	258	3.5%	113	3.5%
	0 (not obstructed)	7169	96.5%	3149	96.5%
Type of warning device at crossing: stopsign	1 (yes)	1190	16%	875	26.8%
	0 (no)	6237	84%	2387	73.2%
Type of warning device at crossing: audible	1 (yes)	3500	47.1%	1123	34.4%
	0 (no)	3927	52.9%	2139	65.6%
Rural area	1 (yes)	3122	42%	2114	64.8%
	0 (no)	4305	58%	1148	35.2%
Age: equal or below 22 years	1 (yes)	1318	17.7%	123	3.8%
	0 (no)	6109	82.3%	3139	96.2%
Age: between 22 and 55 years	1 (yes)	4213	56.7%	2182	66.9%
	0 (no)	3214	43.3%	1080	33.1%
Age: 55 years or above	1 (yes)	1896	25.5%	957	29.3%
	0 (no)	5531	74.5%	2305	70.7%
Gender	1 (male)	4250	57.2%	3132	96%
	0 (female)	3177	42.8%	130	4%

**Table 3:** Results of truck model (fatality).

Mixed Logit Model						
*AIC*	4285.6					
*BIC*	4407.4					
McFadden Pseudo R-squared	0.41					
Log likelihood funciton	−2122.8					
Number of Observations	3262					
**Variables Description**	**Coefficient**	**Standard Error**	**z**	**Prob.** |z| > Z*	**Elasticity**	
					**Fatality**	**Injury**
Defined for Fatality						
constant	−5.69759	0.42083	−13.54	0		
Vehicle & Train Characteristics						
Vehicle Speed	0.95016	0.56382	1.69	0.0919	1.1%	−0.1%
Train Speed	1.18156	0.19738	5.99	0	15%	−0.4%
Truck indicator in crash	1.13924	0.17476	6.52	0	29%	−2.3%
Crash specific characteristics						
Position of vehicle: vehicle moving over crossing	1.41412	0.25286	5.59	0	84.1%	−5.4%
Circumstances of crash: rail equipment struck highway user	0.77359	0.30060	2.57	0.0101	66%	−3.1%
Driver’s Characteristics						
Age of driver: above 55 years	0.39994	0.17332	2.31	0.0210	10.9%	−0.7%
Action of highway user: motorist went around gate	0.74689	0.28061	2.66	0.0078	3.7%	−0.4%
Highway-rail Grade Crossing Attributes						
Rural area	0.55404	0.20024	2.77	0.0057	33.8%	−1.8%

**Table 4:** Results of truck model (injury).

Mixed Logit Model						
*AIC*	4285.62					
*BIC*	4407.44					
Log likelihood funciton	−2122.8					
McFadden Pseudo R-squared	0.41					
Number of Observations	3262					
**Variables Description**	**Coefficient**	**Standard Error**	**z**	**Prob.** |z| > Z*	**Elasticity**	
					**Fatality**	**Injury**
Defined for Injury						
Constant	−2.89124	0.14348	−20.15	0		
Vehicle & Train Characteristics						
Vehicle Speed	0.80110	0.37177	2.15	0.0312	−0.4%	0.6%
Train Speed	0.43865	0.14474	3.03	0.0024	−1.4%	3.9%
Truck indicator in crash	1.05641	0.12219	8.65	0	−8.4%	17.3%
Driver’s Characteristics						
Motorist behavior: motorist went around gate	0.84825	0.23295	3.64	0.0003	−1.3%	2.9%
Highway-rail Grade Crossing Attributes						
Open space	0.52061	0.12097	4.30	0	−4.0%	10.2%
Rural area	0.28768	0.12235	2.35	0.0187	−3.9%	12.5%
Indicator for primary obstruction of track view	−0.98399	0.33176	−2.97	0.0030	0.4%	−2.8%
Type of warning device at crossing: audible	−0.44218	0.12600	−3.51	0.0004	2.2%	−11%
Highwaynear500 ft (random)	−0.52023 (1.27442)	0.28595 (0.53560)	−1.82 (2.38)	0.0689 (0.0173)	−1.1%	9.8%

**Table 5:** Results of auto model (fatality).

Mixed Logit Model						
*AIC*	11,803.51					
*BIC*	11,955.58					
McFadden Pseudo R-squared	0.28					
Log likelihood	−5879.75					
Number of Observations	7427					
**Variables Description**	**Coefficient**	**Standard Error**	**z**	**Prob.** |z| > Z*	**Elasticity**	
					**Fatality**	**Injury**
Defined for Fatality						
constant	−2.76603	0.11382	−24.30	0		
Vehicle & Train Characteristics						
Vehicle Speed > 50 mph	1.26589	0.27959	4.53	0	1.6%	−0.4%
Train Speed > 50 mph	1.96896	0.1900	16.55	0	16.8%	−0.6%
Crash specific characteristics						
Position of highway user: stalled or stuck on crossing	−1.75671	0.26037	−6.75	0	−25%	0.5%
Driver’s Characteristics						
Age of driver: above 55 years	0.84202	0.10222	8.24	0	18.5%	−2.4%
Action of highway user: stopped and then proceeded	−1.05055	0.27969	−3.76	0.0002	−5.7%	0.2%
Action of highway user: went around gate	0.79343	0.12635	6.28	0	9.6%	−1.7%
Action of highway user: stopped on crossing(random)	−1.09872 (1.13415)	0.50617 (0.63517)	−2.17 (1.79)	0.0300 (0.0742)	−1.1%	0.3%
Driver’s gen: male	−0.31985	0.09751	−3.28	0.0010	−16.6%	1.4%
Highway-rail Grade Crossing Attributes						
Rural area	0.75470	0.10080	7.49	0	27.8%	−3.2%
Type of warning device: stopsign	0.39611	0.12977	3.05	0.0023	5.6%	−0.6%

**Table 6:** Results of auto model (injury).

Mixed Logit Model						
Log likelihood	−5812.13410					
McFadden pseudo R-squared	0.29					
Number of Observations	7427					
**Variables Description**	**Coefficient**	**Standard Error**	**z**	**Prob.** |z| > Z*	**Elasticity**	
					**Fatality**	**Injury**
Defined for Injury						
Constant	−1.56308	0.06568	−23.80	0		
Vehicle & Train Characteristics						
Vehicle Speed > 50 mph	0.47109	0.20966	2.25	0.0246	−0.3%	0.4%
Train Speed > 50 mph	0.40962	0.08969	4.57	0	−1.4%	3.5%
Crash specific characteristics						
Position of highway user: move over crossing	0.98392	0.08421	11.68	0	−23.7%	37.5%
Driver’s Characteristics						
Age of driver: above 55 years	0.18934	0.0619	3.06	0.0022	−1.5%	3.3%
Driver’s gender: male	−0.38730	0.05430	−7.13	0	6.2%	−15.9%
Action of highway user: went around gates	0.62722	0.09651	6.5	0	−3.7%	5.1%
Action of highway user: did not stop	0.20730	0.07954	2.61	0.0092	−2.8%	4.4%
Highway-rail Grade Crossing Attributes						
Rural area	0.29927	0.0564	5.31	0	−4.1%	8.4%
Type of warning device: stopsign	0.25142	0.07373	3.41	0.0006	−1.4%	2.6%

## Data Availability

Some or all data, models, or code that support the findings of this study are available from the corresponding author upon reasonable request. The FRA data used to support the findings of this study can be downloaded from the website: https://safetydata.fra.dot.gov/OfficeofSafety/default.aspx (accessed on 25 July 2021).
